# No causal effect of tea consumption on cardiovascular diseases: A two-sample Mendelian randomization study

**DOI:** 10.3389/fcvm.2022.870972

**Published:** 2022-09-07

**Authors:** Lu Chen, Xingang Sun, Liangrong Zheng

**Affiliations:** Department of Cardiology and Atrial Fibrillation Center, The First Affiliated Hospital, School of Medicine, Zhejiang University, Hangzhou, China

**Keywords:** tea consumption, coronary artery disease, myocardial infarction, atrial fibrillation, heart failure, Mendelian randomization

## Abstract

**Background:**

Numerous studies have been conducted to investigate the relationship between tea consumption and the risk of cardiovascular diseases (CVD); however, no conclusive results have been achieved. We conducted a Mendelian randomization (MR) study to elucidate the causal associations between tea consumption and several CVD outcomes, including coronary artery disease (CAD), myocardial infarction (MI), atrial fibrillation (AF), and heart failure (HF).

**Methods:**

Independent single-nucleotide polymorphisms (SNPs) genome-wide significantly associated with tea consumption were used as instrumental variables (IVs). Summary statistics for CVD outcomes were obtained from the corresponding genetic consortia and the FinnGen consortium. The inverse-variance weighted (IVW) method was the primary analytical method, and MR estimates from different data sources were combined using fixed-effects meta-analysis. Supplementary MR analyses, including the weighted median, MR-Egger, and the MR pleiotropy residual sum and outlier methods, were conducted to evaluate the robustness of the results. Further MR analyses were repeated by including more genetic variants at a higher *P*-value threshold.

**Results:**

We found that genetically predicted tea consumption was not causally associated with any CVD outcomes in the IVW method using data from large genetic consortia [CAD: odds ratio (OR) = 1.00, 95% confidence interval (CI), 0.91, 1.10, *P* = 0.997; MI: OR = 0.98, 95% CI, 0.90, 1.08, *P* = 0.751; AF: OR = 0.97, 95% CI, 0.92, 1.03, *P* = 0.350; HF: OR = 0.96, 95% CI, 0.88, 1.05, *P* = 0.401] or the FinnGen consortium (CAD: OR = 1.06, 95% CI, 0.96, 1.17, *P* = 0.225; MI: OR = 1.01, 95% CI, 0.89, 1.15, *P* = 0.882; AF: OR = 1.00, 95% CI, 0.88, 1.14, *P* = 0.994; HF: OR = 0.96, 95% CI, 0.88, 1.04, *P* = 0.362). The results were robust and consistent across meta-analysis, supplementary MR analyses, and analyses with more IVs included.

**Conclusion:**

This MR study revealed no causal association between tea consumption and four CVD outcomes, suggesting that tea consumption may not be beneficial for the primary prevention of CVD.

## Introduction

Cardiovascular diseases (CVD) is the leading cause of morbidity and mortality worldwide. It was estimated that 17.9 million people died due to cardiovascular-related complications in 2016 (1), and the burden of CVD in terms of diminished quality of life, loss of productivity, and healthcare costs remains enormous (2). In addition to changing well-known risk factors for CVD (such as obesity, diabetes mellitus, high blood pressure, and high cholesterol) (3), recent studies have focused on modifiable lifestyle factors (such as diet, physical activity, and smoking) (4–6).

As one of the most ancient and commonly consumed beverages globally, tea has attracted tremendous attention for its potential beneficial effects on health (7). Although a number of studies have investigated the association between tea consumption and CVD, there have been no conclusive results. To date, no randomized controlled trial (RCT) has investigated the relationship between tea consumption and risk of CVD outcomes, and only one RCT conducted by Sone et al. assessed whether catechin-enriched green tea consumption affected CVD risk factors (8). Several observational studies have suggested that tea consumption reduces the risk of CVD (7, 9, 10), while other studies have failed to show such an association (11, 12). The conflicting findings from observational studies may be due to differences in the study population, tea consumption assessment methods, small sample sizes, and covariates adjusted in the statistical models (such as socioeconomic status and education levels). In addition, other unmeasured confounding factors and reverse causality inherent in traditional epidemiological studies may make the observed associations uncertain (13). Therefore, it is essential to precisely elucidate the causal associations between tea consumption and CVD outcomes free from confounders and reverse causality.

Although RCTs are the gold standard for assessing causality, well-designed RCTs often consume considerable human, financial, and time resources. The Mendelian randomization (MR) approach, a natural genetic counterpart of RCT, has been widely applied in disease epidemiology to investigate the causal relationship between exposures and outcomes. This approach uses genetic variants that have a specific effect on a trait (exposure) as instrumental variables (IVs), with those who inherit or do not inherit the genetic variant assigned to a higher or lower dosage of the specific trait. As it was assumed that genetic variants were unlikely to be affected by disease status and were randomly allocated at meiosis, the MR method avoids confounding factors and reverse causation (14). Given the advantages of the MR approach, we utilized genetic variants that influence tea consumption as IVs to determine the associations between tea consumption and the risk of several CVD outcomes, including coronary artery disease (CAD), myocardial infarction (MI), atrial fibrillation (AF), and heart failure (HF) in a two-sample MR design.

## Materials and methods

### Study design

**FIGURE 1:**
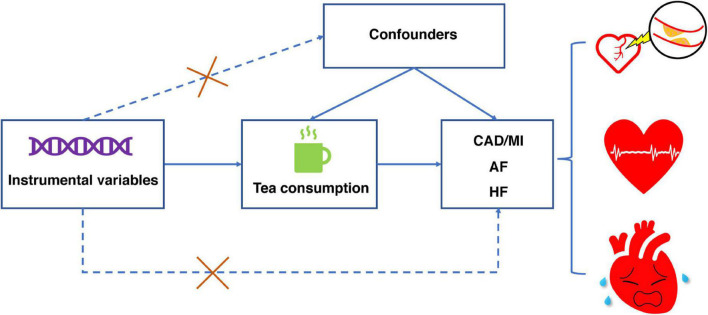
Design of Mendelian randomization study of tea consumption and risk of cardiovascular diseases. This Mendelian randomization study builds upon the assumptions that instrumental variables are associated with tea consumption but not with confounders, and that instrumental variables affect the risk of the four cardiovascular diseases only through tea consumption. CAD, coronary artery disease; MI, myocardial infarction; AF, atrial fibrillation; HF, heart failure.

This study used a two-sample MR design to estimate the causal inferences between tea consumption and several CVD outcomes (Figure 1) and followed the Strengthening the Reporting of Observational Studies in Epidemiology Using Mendelian Randomization (STROBE-MR) statement (15). The MR study was based on the following three assumptions. First, the genetic variants selected as IVs were strongly associated with exposure (tea consumption). Second, the IVs were not associated with any confounders. Third, the IVs directly affected the outcomes (CVD) through exposure rather than other pathways.

### Outcome data sources

Summary statistics for CAD and MI were obtained from the Coronary ARtery DIsease Genome-wide Replication and Meta-analysis (CARDIoGRAM) plus The Coronary Artery Disease (C4D) Genetics (CardiogramplusC4D) consortium (16). The study included 60,801 patients with CAD (among whom there were 43,676 MI cases) and 123,504 controls. Cases were defined using a broad definition of CAD, including MI, acute coronary syndrome, chronic stable angina, or coronary artery stenosis greater than 50%. As for AF, we obtained summary-level data from the genome-wide association studies (GWAS) meta-analysis conducted by Nielsen et al., with case status defined as paroxysmal or permanent AF or atrial flutter (17). For HF, summary-level data were extracted from the largest GWAS meta-analysis among European individuals performed by the Heart Failure Molecular Epidemiology for Therapeutic Targets (HERMES) Consortium (18). We also obtained summary statistics for CVD outcomes from the FinnGen study (release 6) for replication purposes (19), with the number of cases ranging from 15,787 for MI to 30,098 for HF. Detailed descriptions of the outcome data sources are presented in Table 1.

**TABLE 1:** Descriptions for data sources of CVD outcomes involved in this MR study.

Outcome traits	Data sources	Cases	Controls	Sample overlap*	Ancestry	Covariates adjusted in GWAS	Access link
CAD	CARDIoGRAMplusC4D (16)	60,801	123,504	0	Mixed	Age, sex, and PCs	http://www.cardiogramplusc4d.org
CAD	FinnGen consortium (19)	25,707	234,698	0	European	Age, sex, 10 PCs, and genotyping batch	https://www.finngen.fi/en, phenocode: I9_CHD
MI	CARDIoGRAMplusC4D (16)	43,676	128,199	0	Mixed	Age, sex, and PCs	http://www.cardiogramplusc4d.org
MI	FinnGen consortium (19)	15,787	222,551	0	European	Age, sex, 10 PCs, and genotyping batch	https://www.finngen.fi/en, phenocode: I9_MI
AF	Nielsen et al. (17)	60,620	970,216	349,376 (33.9%)	European	Birth year, sex, genotype batch, and 1–4 PCs	http://csg.sph.umich.edu/willer/public/afib2018
AF	FinnGen consortium (19)	28,670	135,821	0	European	Age, sex, 10 PCs, and genotyping batch	https://www.finngen.fi/en, phenocode: I9_AF
HF	HERMES (18)	47,309	930,014	349,376 (35.7%)	European	Age and sex, and PCs in individual studies where applicable	https://cvd.hugeamp.org/dinspector.html?dataset=GWAS_HERMES_eu
HF	FinnGen consortium (19)	30,098	229,612	0	European	Age, sex, 10 PCs, and genotyping batch	https://www.finngen.fi/en, phenocode: I9_HEARTFAIL_ALLCAUSE

CVD, cardiovascular diseases; MR, Mendelian randomization; GWAS, genome-wide association studies; CAD, coronary artery disease; MI, myocardial infarction; AF, atrial fibrillation; HF, heart failure; CARDIoGRAMplusC4D, Coronary ARtery DIsease Genome-wide Replication and Meta-analysis (CARDIoGRAM) plus The Coronary Artery Disease (C4D) Genetics; HERMES, Heart Failure Molecular Epidemiology for Therapeutic Targets. PCs, principal components.

*The overlapping sample size (if all overlapping samples appeared in the genome-wide association studies of the exposure and outcome) was divided by the larger sample size of the corresponding outcome trait and tea consumption.

### Instrumental single-nucleotide polymorphisms selection

**FIGURE 2:**
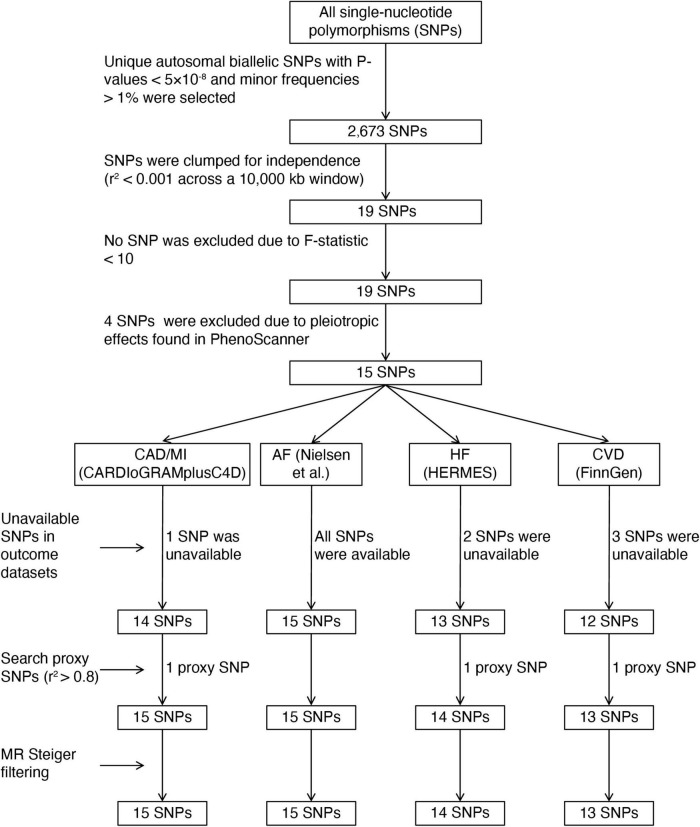
The flowchart of instrumental variables selection. CAD, coronary artery disease; MI, myocardial infarction; AF, atrial fibrillation; HF, heart failure; CVD, cardiovascular diseases; CARDIoGRAMplusC4D, Coronary ARtery DIsease Genome-wide Replication and Meta-analysis (CARDIoGRAM) plus The Coronary Artery Disease (C4D) Genetics; HERMES, Heart Failure Molecular Epidemiology for Therapeutic Targets.

Similar to a previous MR study (20), we identified tea consumption-associated single-nucleotide polymorphisms (SNPs) based on the summary-level statistics for untransformed daily cups of tea consumption (Phenotype Code:1488_raw) obtained from GWAS published by the Neale laboratory (GWAS round 2),^1^ including up to 349,376 individuals of European descent. The covariates adjusted in the GWAS for tea intake were the first 20 principal components + sex + age + age^2^ + sex × age + sex × age^2^. Tea intake was obtained at baseline from a dietary questionnaire of “How many cups of tea do you drink each day? (Include black and green tea).” The overall workflow of SNP selection is summarized in Figure 2. We selected autosomal biallelic SNPs with *P*-values < 5 × 10^–8^ and minor frequencies > 1%, leaving 2,673 unique SNPs. We then performed clumping function using the TwoSampleMR R package (version 0.5.6) to select genetic variants without any linkage disequilibrium (LD) (*r*^2^ < 0.001 across a 10,000 kb window) based on European sample reference data from the 1,000 Genomes Project (21). Finally, 19 independent SNPs associated with tea consumption (*P* < 5 × 10^–8^) remained; detailed information on these SNPs is shown in Supplementary Table 1. We further proceeded with the following steps to determine valid IVs: First, the F-statistic was computed to measure the strength of the IVs. The F-statistic of each SNP was > 10 (Supplementary Table 1), indicating a low risk of weak instrument bias; none of these SNPs was excluded in this step (22). Second, we searched for SNPs in the PhenoScanner database^2^ to assess whether these SNPs were associated with established CVD risk factors (*P* < 5 × 10^–8^), such as obesity (23, 24), systolic and diastolic blood pressure (25), lipid traits (26), type 2 diabetes mellitus (27), smoking, and alcohol intake (28). A total of four SNPs showed horizontal pleiotropic effects and were excluded from the MR analyses (Supplementary Table 2). Third, SNPs not available in the outcome datasets were replaced by proxy SNPs in high LD (*r*^2^ > 0.8) by searching an online website^3^ (Supplementary Table 3). Fourth, we applied MR Steiger filtering to test the causal direction of each extracted SNP on the exposure and the outcome. This approach calculated the variance explained in the exposure and outcome by the IV and tested whether the variance in the outcome was less than the exposure. No SNP was removed for false causal direction, which showed evidence of primarily affecting outcomes rather than the exposure.

### Statistical analysis

Our primary MR analysis was performed using the multiplicative random-effects inverse-variance weighted (IVW) method, which could provide a robust causal estimate in the presence of heterogeneity (29). The fixed-effects meta-analysis method was applied to combine MR estimates derived from two different data sources. We also performed several supplementary MR methods to evaluate the robustness of the results, including the weighted median (30), MR-Egger regression (31), and MR-pleiotropy residual sum and outlier (MR-PRESSO) (32). The weighted median method can provide consistent causal estimates when more than 50% of the weight in the analysis is derived from valid IVs (30). MR-Egger regression was applied to detect horizontal pleiotropy with *P*-value for its intercept and generate estimates after correcting for pleiotropy (31). The MR-PRESSO test can detect and correct horizontal pleiotropic outliers in the IVW method and explore significant differences in the causal assessments before and after excluding outliers (32). Cochran’s Q statistic and I^2^ statistic were used to evaluate the heterogeneity among SNPs, with Cochran’s Q derived *P*-value < 0.05 or I^2^ > 25% considered as horizontal pleiotropy. Furthermore, we performed the leave one-out analysis to determine whether any pleiotropic SNPs drove MR estimates. Finally, considering the low number of IVs (*P* < 5 × 10^–8^) in the primary MR analysis, which explained the low variance (approximately 0.37%) in tea consumption, we added genetic variants with higher *P*-values for tea consumption (*P* < 5 × 10^–7^) and then repeated the MR analyses stated above to investigate whether the results were robust. A similar selection of IVs with *P*-values < 5 × 10^–7^ is shown in Supplementary Figure 1.

Power calculations were performed to evaluate the required effects of exposure on the outcomes at 80% power based on the sample size of each outcome and variance of exposure explained by the IVs on a web-based application.^4^ The results are presented in (Supplementary Table 4).

Results are presented as odds ratios (ORs), and all reported ORs and corresponding 95% confidence intervals (CIs) of CVD were scaled to per cup increment in daily tea consumption. A Bonferroni-corrected *P*-value of < 0.013 (correcting for four outcomes) was considered as the threshold of significance. All MR analyses were performed using the TwoSampleMR (33) and MRPRESSO (32) packages in R (version 4.0.2).

## Results

**FIGURE 3:**
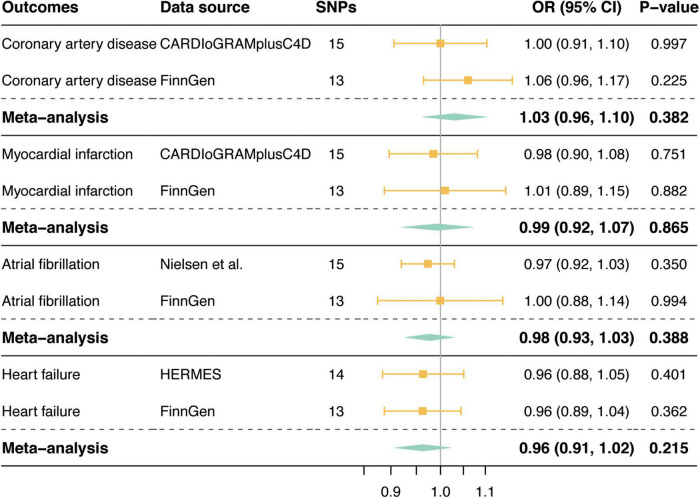
Mendelian randomization associations of tea consumption with risk of cardiovascular diseases. SNPs, single nucleotide polymorphisms; OR, odds ratio; CI, confidence interval; CARDIoGRAMplusC4D, Coronary ARtery DIsease Genome-wide Replication and Meta-analysis (CARDIoGRAM) plus The Coronary Artery Disease (C4D) Genetics; HERMES, Heart Failure Molecular Epidemiology for Therapeutic Targets.

Figure 3 shows the associations between genetically determined tea consumption (per cup increment in daily tea consumption) and the risk of CVD outcomes in the primary MR analyses. We observed no causal association between tea consumption and CVD outcomes in the GWAS of large genetic consortia (CAD: OR = 1.00, 95% CI, 0.91, 1.10, *P* = 0.997; MI: OR = 0.98, 95% CI, 0.90, 1.08, *P* = 0.751; AF: OR = 0.97, 95% CI, 0.92, 1.03, *P* = 0.350; HF: OR = 0.96, 95% CI, 0.88, 1.05, *P* = 0.401; Figure 3) or in the FinnGen consortium (CAD: OR = 1.06, 95% CI, 0.96, 1.17, *P* = 0.225; MI: OR = 1.01, 95% CI, 0.89, 1.15, *P* = 0.882; AF: OR = 1.00, 95% CI, 0.88, 1.14, *P* = 0.994; HF: OR = 0.96, 95% CI, 0.88, 1.04, *P* = 0.362; Figure 3). Meta-analysis results combining MR estimates from different data sources also revealed no causal inference between tea consumption and CVD (*P*-values for all CVD outcomes were > 0.05; Figure 3).

Supplementary MR analyses, including the weighted median and MR-Egger methods, also showed no association between genetically predicted tea consumption and any CVD outcomes (Table 2). There was low to moderate heterogeneity detected in several analyses indicated by P_Cochran’*s Q*_ and *I*^2^ statistics (CAD in the CARDIoGRAMplusC4D: P_Cochran’*s Q*_ = 0.097, *I*^2^ = 33.9%; AF in the FinnGen: P_Cochran’*s Q*_ = 0.147, *I*^2^ = 29.7%; HF in the HERMES: P_Cochran’*s Q*_ = 0.052, *I*^2^ = 41.5%; Table 2). Nevertheless, the multiplicative random-effects IVW performed in primary MR analyses could provide a robust causal estimate even in the presence of heterogeneity. Moreover, the *P*-values for the intercept in the corresponding MR-Egger regression were all above 0.05 (Table 2), suggesting no evidence of pleiotropy. MR-PRESSO only identified one outlier SNP (rs4410790) for CAD in CARDIoGRAMplusC4D (Table 2). After excluding this outlier, there was no evidence of causal association between genetically determined tea consumption and CAD (OR = 0.92, 95% CI, 0.85, 1.00, *P* = 0.078, Table 2). Furthermore, we performed leave-one-out analyses of all the CVD outcomes from different data sources. The results consistently indicated that no single SNP dramatically biased the non-causal associations between tea consumption and CVD (Supplementary Figures 2, 3).

**TABLE 2:** Supplementary analyses of the associations between tea consumption and cardiovascular diseases.

Outcomes	Data source	SNPs	P_Cochran’s Q_	I^2^ (%)	Weighted median method		MR-Egger regression			MR-PRESSO			
							
					OR (95% CI)	P	OR (95% CI)	P	P_intercept_	OR (95% CI)	P	P_global test_	Outlier
CAD	CARDIoGRAMplusC4D	15	0.097	33.9	0.99 (0.87, 1.12)	0.837	1.18 (0.96, 1.45)	0.130	0.093	0.92 (0.85, 1.00)	0.078	0.033	rs4410790
	FinnGen	13	0.520	0.0	1.07 (0.94, 1.23)	0.295	1.21 (0.98, 1.49)	0.102	0.190	–*	–*	0.508	0
MI	CARDIoGRAMplusC4D	15	0.353	9.0	0.93 (0.81, 1.06)	0.288	1.09 (0.88, 1.34)	0.437	0.310	–*	–*	0.130	0
	FinnGen	13	0.282	16.1	1.03 (0.88, 1.21)	0.720	1.24 (0.96, 1.59)	0.124	0.099	–*	–*	0.314	0
AF	Nielsen et al.	15	0.450	0.0	0.98 (0.90, 1.06)	0.554	1.08 (0.96, 1.22)	0.240	0.081	–*	–*	0.390	0
	FinnGen	13	0.147	29.7	1.07 (0.93, 1.24)	0.349	1.24 (0.96, 1.61)	0.121	0.082	–*	–*	0.167	0
HF	HERMES	14	0.052	41.5	0.99 (0.91, 1.08)	0.883	1.01 (0.84, 1.22)	0.900	0.568	–*	–*	0.090	0
	FinnGen	13	0.496	0.0	0.97 (0.86, 1.09)	0.594	1.01 (0.85, 1.21)	0.904	0.551	–*	–*	0.454	0

SNPs, single nucleotide polymorphisms; MR-PRESSO indicates MR-pleiotropy residual sum and outlier; OR, odds ratio; CI, confidence interval; CAD, coronary artery disease; MI, myocardial infarction; AF, atrial fibrillation; HF, heart failure; CARDIoGRAMplusC4D, Coronary ARtery DIsease Genome-wide Replication and Meta-analysis (CARDIoGRAM) plus The Coronary Artery Disease (C4D) Genetics; HERMES, Heart Failure Molecular Epidemiology for Therapeutic Targets; *the result was the same as the inverse-variance weighted method due to no outlier detected.

When we lowered the *P*-value threshold of IV selection (*P* < 5 × 10^–7^), tea consumption was not significantly associated with CVD outcomes in the random-effects IVW method or in the fixed-effect meta-analysis combining MR estimates from different data sources (Supplementary Figure 4), which was in line with the results of the primary analyses. Likewise, the relationships between tea consumption and CVD outcomes were consistent across the weighted median and MR-Egger regression methods, with modest heterogeneity observed in several analyses. No evidence of pleiotropy was indicated by the intercept from the MR-Egger regression (All P_intercept_ were > 0.05, Supplementary Table 5). The MR-PRESSO method identified one outlier (rs2117137) for AF in the GWAS meta-analysis by Nielsen et al., and the non-causal association remained after excluding this outlier (Supplementary Table 5).

## Discussion

This MR study demonstrated no causal relationship between tea consumption and several CVD outcomes, including CAD, MI, AF, and HF, thus providing little rationale for drinking tea to reduce CVD risk.

Over the last 20 years, many cross-sectional and prospective studies have investigated the relationships between tea consumption and CVD; however, no definite conclusions can be drawn from available data. In a multi-ethnic study involving 6,508 participants, Miller et al. found a statistically significant lower incidence of cardiovascular events in ≥ 1 cup/day tea drinkers (adjusted hazard ratio, 0.71; 95% CI, 0.53, 0.95) (34). A similar inverse association was observed between tea consumption and CVD mortality (35), risk of coronary heart disease (CHD) (36), incident MI (37), and both paroxysmal AF and persistent AF (38). Several studies have suggested that there was no correlation between tea consumption and the risk of developing CVD or CHD (39, 40). In addition, divergent results were obtained for different types of tea. For example, in a study by Mineharu et al. (41), black tea consumption was not associated with CVD-related mortality. In contrast, green tea and oolong tea reduced the mortality risk of CVD. Another meta-analysis, including 13 studies on black tea and five studies on green tea, suggested that green tea consumption was significantly associated with a decreased risk of CAD; on the contrary, black tea consumption did not show such an inverse association (42).

In this MR study, we found no protective effect of tea consumption on comprehensive CVD, which corroborated the results of some traditional observational studies and extended the findings of a previous MR study of tea consumption on the risk of stroke (20). The lack of causality in this MR study indicated that the protective effect of tea consumption on CVD observed in several observational studies may be limited by confounders and reverse causation rather than by identifying a causal correlation. It is worth noting that these epidemiological studies are potentially biased by confounding factors, such as different lifestyles between tea drinkers and non-tea drinkers, baseline flavonoid intake, background health, socioeconomic status, and many other factors (10). Another explanation for the divergent results is that the contents of chemical compounds differ in different types of tea. Tea is rich in polyphenols, particularly flavonoids, and green tea is more abundant than black or oolong tea (43). Catechins, a distinctive polyphenolic compound that can improve redox status, inhibit inflammation, reduce platelet aggregation, and ameliorate hyperlipidemia, constitute 80–90% and 20–30% of total flavonoids in green tea and black tea, respectively (42, 44). Thus, black tea, which is mainly consumed in Europe, may have fewer protective components than green tea, which is principally consumed in Asia (45). As a result, the preference for black tea in the European participants in our study might be the cause of the unbeneficial effect of tea consumption on CVD, although we used summary statistics for tea consumption, including both green tea and black tea. In addition, the null association between tea consumption and CVD risk in our MR study might be due to the balanced impact of cardioprotective and cardio-detrimental components of tea and dietary components. Therefore, further MR studies are warranted to determine the causal effects of different tea subtypes on CVD.

This study has several strengths. First, for the first time, we applied the MR method to investigate the causal associations between tea consumption and the risk of comprehensive CVD outcomes, avoiding potential confounding factors and reverse causation. Second, we utilized summary-level data from several large genetic consortia and the FinnGen consortium, and the results of the different datasets were consistent, assuring the reliability of our findings. Third, our results were less likely to be biased by population stratification, as the analyses were restricted to individuals of European ancestry. Nonetheless, this might limit the generalizability of our conclusions to other ethnicities.

However, this study has potential limitations. First, as mentioned above, the contents of chemical compounds in different types of tea are different, and the commonly consumed types also differ worldwide. Thus, our results might be biased because we could not assess whether the effects of different kinds of tea (black tea or green tea) on CVD risk were similar owing to a lack of detailed information. Second, data on tea consumption were obtained from a dietary questionnaire, which may be susceptible to measurement errors. Third, potential pleiotropy is a major limitation of MR studies. However, we used several sensitivity analyses that were more robust to pleiotropy to minimize the bias from pleiotropy, and the results remained consistent. Fourth, the required ORs of tea consumption on CVD outcomes to achieve 80% statistical power ranged from 0.621 (or 1.399) to 0.811 (or 1.193) (Supplementary Table 4). However, our estimated effect sizes did not reach the threshold to achieve 80% statistical power, indicating that there might be insufficient statistical power to detect the weak associations between tea consumption and CVD outcomes. It might be due to the low variance explained by the SNPs at the genome-wide significance level (approximately 0.37%). In such circumstances, we should be cautious with interpreting the negative results as there remains the possibility that false-negative results may occur. Therefore, we further investigated whether the MR estimates were robust when more genetic variants were selected as IVs, and the results remained consistent. However, increasing the number of genetic variants increases the chance of weak instrument bias to some extent. We used the F-statistic to assess instrument strength and found that there was no weak instrument (*F* > 10 for each variant). Fifth, there was some sample overlap in the GWAS of tea consumption and the GWAS meta-analysis by Nielsen et al. for AF and GWAS by HERMES for HF (Table 1), leading to some MR estimate bias. However, the F statistic for each IV was sufficiently large, suggesting that our MR estimates were unlikely to be affected by sample overlap bias (46). Furthermore, this sample overlap would not lead to bias if genetic associations with tea consumption were estimated in non-cases only (as is usual for continuous phenotypes) (46). Moreover, MR estimates obtained from the FinnGen consortium (no sample overlap) yielded similar results. Sixth, since we used summary-level data in our MR analyses, we cannot rule out the possibility of a non-linear causal relationship between tea consumption and CVD risk. The potential dose–response causal associations between tea consumption and CVD risk should be evaluated in further MR studies with individual-level data and longitudinal designs.

## Conclusion

In conclusion, this MR study found no causal association between tea consumption and several CVD outcomes, including CAD, MI, AF, and HF, suggesting that tea consumption may not benefit the primary prevention of CVD.

## Data availability statement

The original contributions presented in this study are included in the article/Supplementary material, further inquiries can be directed to the corresponding author.

## Ethics statement

Ethics approval or informed consent was not required since this study was based on publicly available databases.

## Author contributions

LC and LZ designed the study. LC and XS analyzed the data, prepared the original draft, and revised and edited the manuscript. LZ supervised the study and acquired funding for the work. All authors have read and agreed to the published version of the manuscript.

## Footnotes

1
http://www.nealelab.is/uk-biobank

2
http://www.phenoscanner.medschl.cam.ac.uk

3
http://snipa.helmholtz-muenchen.de/snipa3/

4
http://cnsgenomics.com/shiny/mRnd/
