# Supplementary material for: No causal effect of tea consumption on cardiovascular diseases: A two-sample Mendelian randomization study
Source: Front Cardiovasc Med. 2022 Sep 7;9:870972. doi: 10.3389/fcvm.2022.870972 (PMC9491345; doi:10.3389/fcvm.2022.870972)
Supplement: Supplementary file 1 [file Data_Sheet_1.pdf]

Supplementary Material

1 Supplementary Figures and Tables

1.1 Supplementary Figures

Supplementary Figure 1. The flowchart of selection of instrumental variables with P-values < 5×10^{-7}.

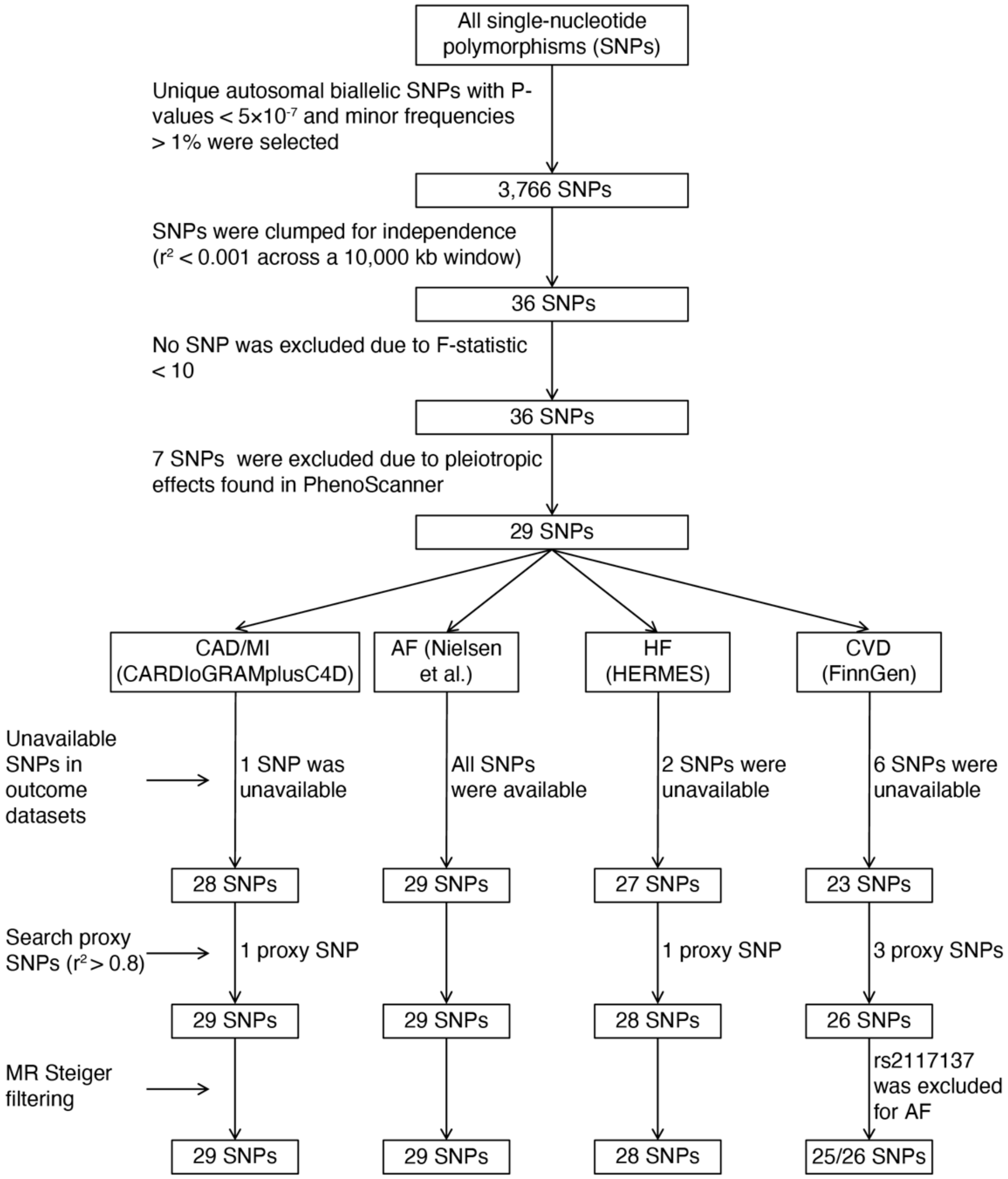

CAD, coronary artery disease; MI, myocardial infarction; AF, atrial fibrillation; HF, heart failure; CVD, cardiovascular disease; CARDIoGRAMplusC4D, Coronary ARtery DIsease Genome-wide Replication and Meta-analysis (CARDIoGRAM) plus The Coronary Artery Disease (C4D) Genetics; HERMES, Heart Failure Molecular Epidemiology for Therapeutic Targets.

Supplementary Figure 2. Leave-one-out analysis of tea consumption on A) coronary artery disease, B) myocardial infarction, C) atrial fibrillation, and D) heart failure in the corresponding genetic consortia.

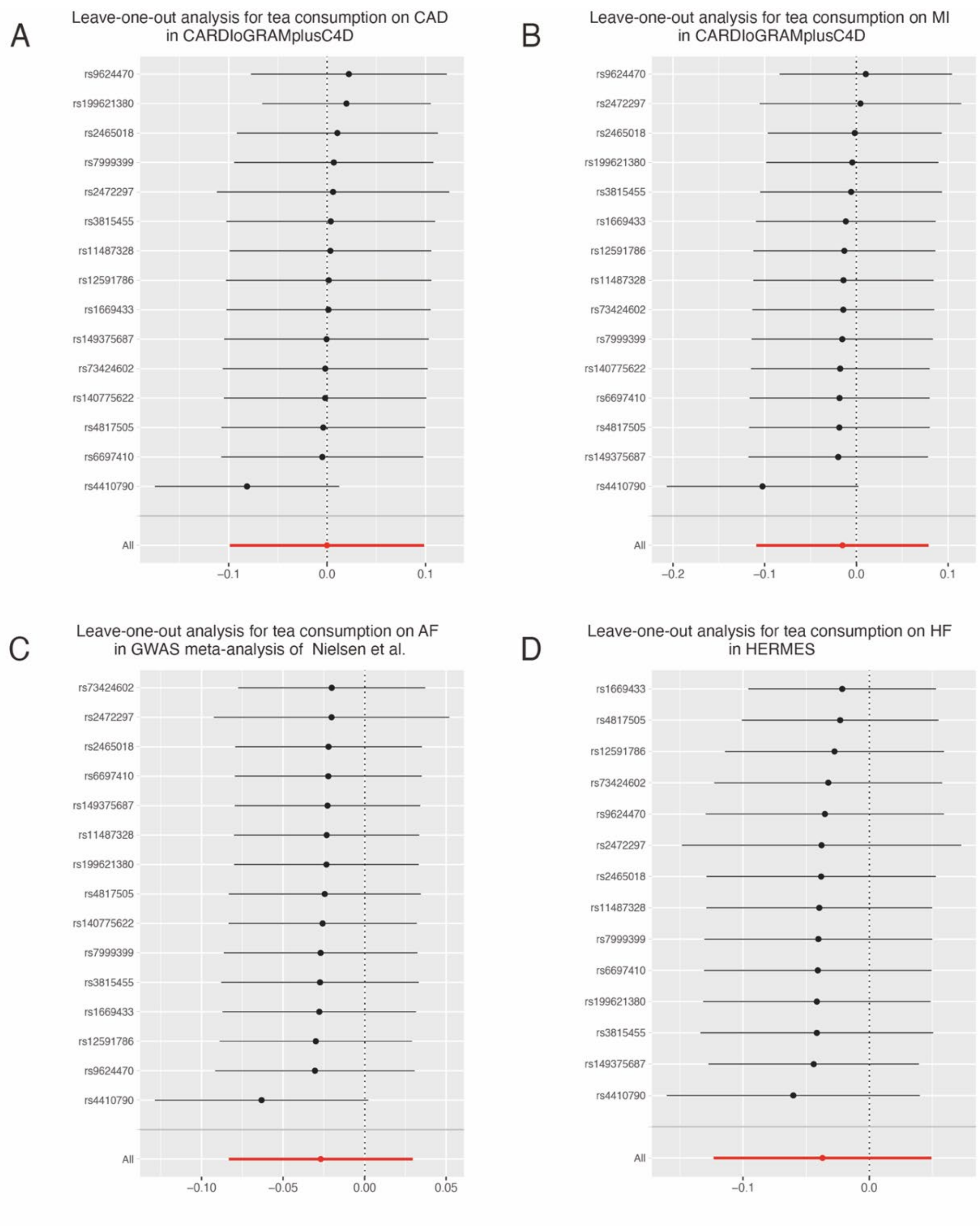

CAD, coronary artery disease; MI, myocardial infarction; AF, atrial fibrillation; HF, heart failure; CARDIoGRAMplusC4D, Coronary ARtery DIsease Genome-wide Replication and Meta-analysis

(CARDIoGRAM) plus The Coronary Artery Disease (C4D) Genetics; HERMES, Heart Failure Molecular Epidemiology for Therapeutic Targets; GWAS, genome-wide association studies.

Supplementary Figure 3. Leave-one-out analysis of tea consumption on A) coronary artery disease, B) myocardial infarction, C) atrial fibrillation, and D) heart failure in the FinnGen consortium.

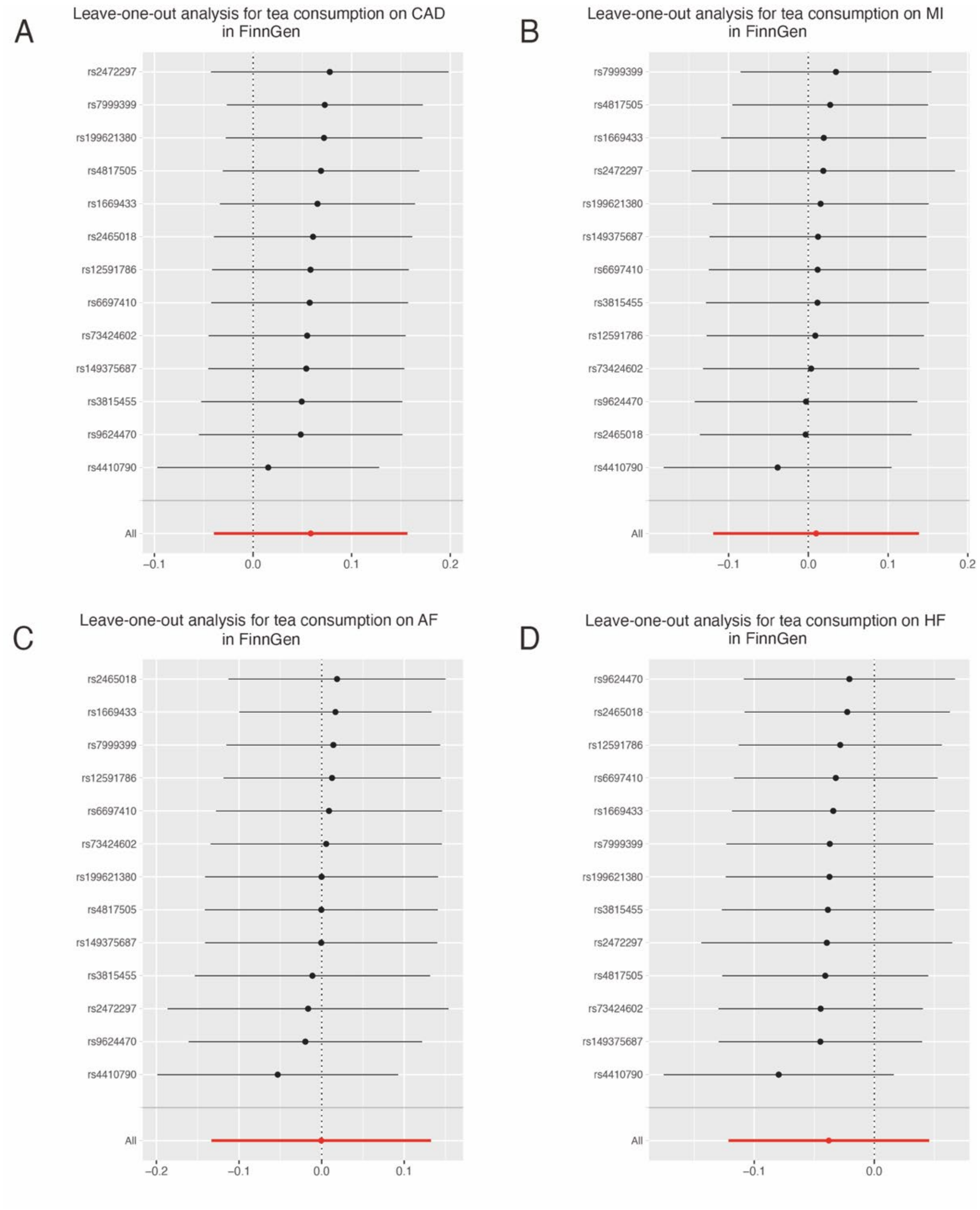

CAD, coronary artery disease; MI, myocardial infarction; AF, atrial fibrillation; HF, heart failure.

Supplementary Figure 4. Mendelian randomization associations of tea consumption with risk of cardiovascular diseases with instrumental variables selected at P-value threshold of 5×10^{-7}.

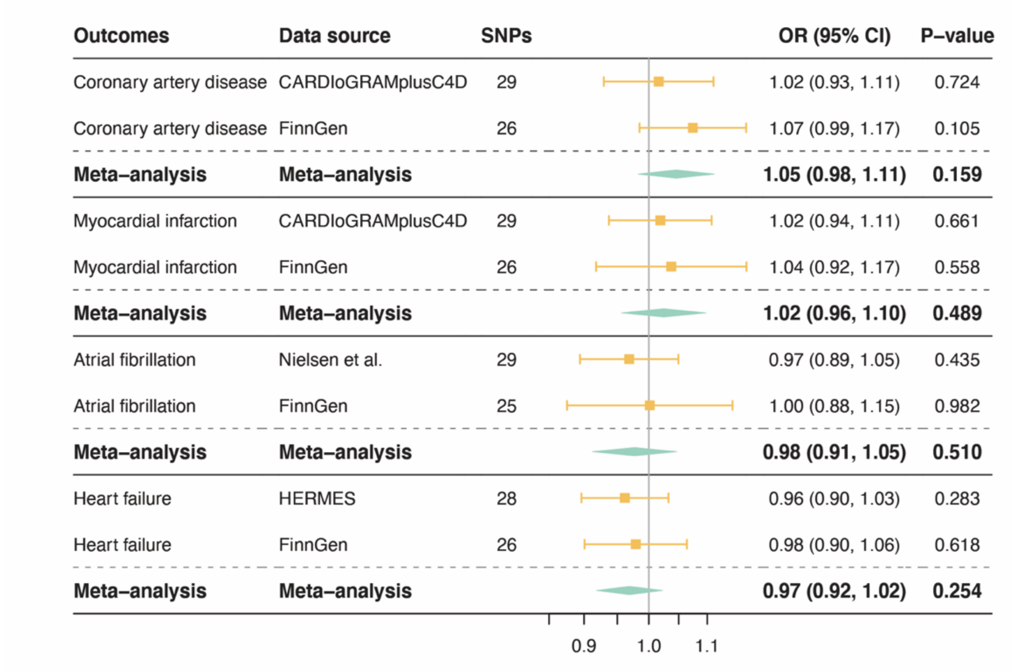

SNPs, single nucleotide polymorphisms; OR, odds ratio; CI, confidence interval; CARDIoGRAMplusC4D, Coronary ARtery DIsease Genome-wide Replication and Meta-analysis (CARDIoGRAM) plus The Coronary Artery Disease (C4D) Genetics; HERMES, Heart Failure Molecular Epidemiology for Therapeutic Targets.

1.2 Supplementary Tables

Supplementary Table 2. Association of potential pleiotropic SNPs with established risk factors for cardiovascular diseases.

P-value cut-off	SNPs	EA	NEA	Trait	Beta	P	PMID
5.00E-08	rs1481012	A	G	Cholesterol ldl	NA	2.00E-15	22331829
5.00E-08	rs11022751	C	T	Body mass index	-0.016	4.19E-09	UKBB
		C	T	Diastolic blood pressure	-0.016	1.74E-08	UKBB
5.00E-08	rs2315024	A	T	Self-reported high cholesterol	-0.005	4.02E-09	UKBB
5.00E-08	rs12600469	G	T	Weight	-0.012	1.72E-08	UKBB
5.00E-07	rs9937521	C	T	Body mass index	-0.071	2.04E-57	28892062
		C	T	Type II diabetes	-0.128	3.80E-22	26551672
5.00E-07	rs1156954	A	G	Ever smoked	0.008	1.77E-09	UKBB
5.00E-07	rs4757390	A	T	Vascular or heart problems diagnosed by doctor: high blood pressure	0.007	1.65E-11	UKBB
		A	T	Self-reported hypertension	0.007	7.29E-10	UKBB

SNPs, single nucleotide polymorphisms; EA, effect alleles; UKBB, the UK Biobank; NEA, non-effect alleles; ldl, low-density lipoprotein. The reported Beta and P-value were obtained from PhenoScanner v2 on February 10, 2022.

Supplementary Table 3. Proxy search results for tea consumption-associated SNPs which were not available in the outcome datasets.

Outcome traits	Data sources	SNPs	Beta	SE	P-value	EA	NEA	Proxy SNP	r^2
CAD/MI	CARDIoGRAMplusC4D	rs199621380	0.041	0.007	4.53E-09	G	T	rs6678013	0.98
HF	HERMES	rs140775622	0.071	0.010	9.33E-13	T	C	NA	NA
HF	HERMES	rs199621380	0.041	0.007	4.53E-09	G	T	rs6678013	0.98
CAD/MI/AF/HF	FinnGen	rs140775622	0.071	0.010	9.33E-13	T	C	NA	NA
CAD/MI/AF/HF	FinnGen	rs11487328	-0.049	0.007	5.16E-12	C	G	NA	NA
CAD/MI/AF/HF	FinnGen	rs199621380	0.041	0.007	4.53E-09	G	T	rs6678013	0.98
CAD/MI/AF/HF	FinnGen	rs2117137	0.037	0.007	1.42E-07	G	A	rs7637670	0.92
CAD/MI/AF/HF	FinnGen	rs11893458	0.036	0.007	3.79E-07	T	A	rs62183782	0.82
CAD/MI/AF/HF	FinnGen	rs114002124	-0.076	0.015	3.85E-07	A	G	NA	NA

SNPs, single nucleotide polymorphisms; EA, effect alleles; NEA, non-effect alleles; SE, standard error; NA, not available; CARDIoGRAMplusC4D, Coronary ARtery DIsease Genome-wide Replication and Meta-analysis (CARDIoGRAM) plus The Coronary Artery Disease (C4D) Genetics; HERMES, Heart Failure Molecular Epidemiology for Therapeutic Targets.

Supplementary Table 4. Power calculation for two-sample MR analyses of tea consumption on 4 cardiovascular diseases by adopting instrumental variables at the genome-wide significance level.

Outcome traits	Data sources	Outcome category	Sample size	α	K	R^2	Required OR†
Coronary artery disease	CARDIoGRAMplusC4D	Binary	184,305	0.05	0.32989338	0.00374335	0.791 or 1.243
Coronary artery disease	FinnGen	Binary	260,405	0.05	0.0987193	0.00345941	0.699 or 1.320
Myocardial infarction	CARDIoGRAMplusC4D	Binary	171,875	0.05	0.25411491	0.00374335	0.764 or 1.268
Myocardial infarction	FinnGen	Binary	238,338	0.05	0.06623786	0.00345941	0.621 or 1.399
Atrial fibrillation	Nielsen et al.	Binary	1,030,836	0.05	0.05880664	0.00374335	0.811 or 1.193
Atrial fibrillation	FinnGen	Binary	164,491	0.05	0.17429525	0.00345941	0.709 or 1.323
Heart failure	HERMES	Binary	977,323	0.05	0.04840672	0.00359739	0.783 or 1.221
Heart failure	FinnGen	Binary	259,710	0.05	0.1158908	0.00345941	0.720 or 1.299

Note: Power calculation for binary outcomes was performed in https://shiny.cnsgenomics.com/mRnd/ by inputting the number of sample size, α, K, R^2 and the required OR to reach the statistical power of 80%.

Example for power calculation for coronary artery disease in CARDIoGRAMplusC4D: choose the binary outcome and power calculation first. Then input the number of sample size (184,305), α (0.05),

K (0.32989338), R^2 (0.00374335), and OR (0.791 or 1.243). Finally, a statistical power of 80% would be reached.

Abbreviations: α, type-I error rate; K, the proportion of cases in the outcome study; R^2, the variance explained by the tea consumption-associated instrumental variables; OR, odds ratio. CARDIoGRAMplusC4D, Coronary ARtery DIsease Genome-wide Replication and Meta-analysis (CARDIoGRAM) plus The Coronary Artery Disease (C4D) Genetics; HERMES, Heart Failure Molecular Epidemiology for Therapeutic Targets. †: The required OR were the minimal effects of tea consumption on each CVD outcome to achieve 80% statistical power.

Supplementary Table 5. Supplementary analyses of the association between tea consumption and cardiovascular diseases with instrumental variables selected at P-value threshold of 5×10^{-7}.

Outcomes	Data source	SNPs	$P_{Cochran's\ Q}$	I^2 (%)	Weighted median method		MR-Egger regression			MR-PRESSO			
					OR (95% CI)	P	OR (95% CI)	P	$P_{intercept}$	OR (95% CI)	P	$P_{global\ test}$	Outlier
CAD	CARDIoGRAMplusC4D	29	0.020	38.4	1.00 (0.88, 1.13)	0.979	1.10 (0.89, 1.35)	0.376	0.748	-*	-*	0.012	0
CAD	FinnGen	26	0.424	2.7	1.10 (0.97, 1.24)	0.150	1.11 (0.91, 1.34)	0.317	0.419	-*	-*	0.456	0
MI	CARDIoGRAMplusC4D	29	0.269	12.9	0.95 (0.84, 1.08)	0.451	1.02 (0.84, 1.24)	0.824	0.968	-*	-*	0.184	0
MI	FinnGen	26	0.085	28.9	1.04 (0.89, 1.21)	0.611	1.07 (0.81, 1.40)	0.642	0.843	-*	-*	0.113	0
AF	Nielsen et al.	29	4.49E-06	62.3	0.98 (0.91, 1.05)	0.569	1.05 (0.89, 1.25)	0.560	0.819	0.99 (0.94, 1.05)	0.737	<4E-04	rs2117137
AF	FinnGen	25	0.006	46.7	1.04 (0.89, 1.22)	0.593	1.12 (0.83, 1.50)	0.467	0.414	-*	-*	0.009	0
HF	HERMES	28	0.042	34.0	0.99 (0.91, 1.07)	0.744	0.98 (0.83, 1.14)	0.759	0.291	-*	-*	0.058	0
HF	FinnGen	26	0.142	23.2	0.97 (0.86, 1.09)	0.583	0.97 (0.81, 1.17)	0.779	0.949	-*	-*	0.157	0

SNPs, single nucleotide polymorphisms; MR-PRESSO indicates MR-pleiotropy residual sum and outlier; OR, odds ratio; CI, confidence interval; CAD, coronary artery disease; MI, myocardial infarction; AF, atrial fibrillation; HF, heart failure; CARDIoGRAMplusC4D, Coronary ARtery DIsease Genome-wide Replication and Meta-analysis (CARDIoGRAM) plus The Coronary Artery Disease (C4D) Genetics; HERMES, Heart Failure Molecular Epidemiology for Therapeutic Targets; *, the result was the same as the inverse-variance weighted method due to no outlier detected.
